# Giant Calcified Frontal Lobe Metastasis as the Initial Manifestation of Pulmonary Large-Cell Neuroendocrine Carcinoma: Diagnostic and Surgical Insights From Intraoperative Diagnosis

**DOI:** 10.7759/cureus.108807

**Published:** 2026-05-13

**Authors:** Satoshi Horiguchi, Manabu Kurosawa, Yuto Mitsuno, Takeyoshi Tsutsui

**Affiliations:** 1 Department of Neurosurgery, Nagahama City Hospital, Nagahama, JPN; 2 Department of Pathology, Nagahama City Hospital, Nagahama, JPN

**Keywords:** brain tumors (primary or brain metastasis), calcified metastasis, intraoperative frozen section, pulmonary large cell neuroendocrine carcinoma, radiological findings

## Abstract

We present a rare case of pulmonary large-cell neuroendocrine carcinoma (LCNEC) manifesting as a solitary, calcified brain metastasis (CBM) in a 69-year-old woman with no known malignancy or respiratory symptoms. Computed tomography (CT) of the brain revealed a large, heterogeneous left frontal lobe mass with coarse intratumoral calcification and central necrosis. Magnetic resonance imaging (MRI) demonstrated ring-like peripheral enhancement, peritumoral edema, and signal voids on gradient-echo (GRE) sequences, suggesting calcification and/or hemorrhage. A solitary pulmonary nodule was identified in the right upper lobe on preoperative chest CT, and the serum progastrin-releasing peptide (proGRP) level was markedly elevated, suggesting a neuroendocrine tumor. Differential diagnoses included high-grade glioma, particularly oligodendroglioma, based on the lesion's location and calcification. Intraoperatively, the tumor was partially calcified and infiltrated the eloquent cortex, necessitating subtotal resection near the pyramidal tract. Frozen sections revealed a proliferation of atypical large cells with rosette-like structures and nuclear palisading, which are inconsistent with glioma and suggestive of a metastatic neuroendocrine neoplasm. Final pathological examination confirmed metastatic LCNEC. Although CBMs are not exceedingly rare, their imaging features can mimic those of primary brain tumors, potentially delaying diagnosis. This case underscores the importance of considering metastatic LCNEC in the differential diagnosis of solitary calcified brain lesions and highlights the practical impact of intraoperative pathology on surgical decision-making.

## Introduction

Pulmonary large-cell neuroendocrine carcinoma (LCNEC) is a rare and aggressive subtype of lung cancer that accounts for approximately 3% of all pulmonary malignancies [1]. Brain metastasis is a common complication in patients with LCNEC and may be the initial manifestation, particularly in asymptomatic individuals [2,3]. Calcification within brain metastases has been reported in various oncological populations, with an incidence ranging from 7.9% to 9.5% at initial presentation [4,5]. Although calcified brain metastases (CBMs) are rare, solitary calcified lesions in patients without a known primary malignancy remain a diagnostic challenge. CBMs can be misdiagnosed as primary brain tumors due to overlapping radiological features. Because calcification is a well-recognized imaging feature of certain primary brain tumors, particularly oligodendroglioma [6], a solitary calcified brain lesion may lead to diagnostic confusion. Distinguishing metastasis from glioma is clinically important because the surgical strategy and extent of resection may differ between these entities. Here, we report a rare case of a solitary calcified frontal lobe tumor ultimately diagnosed as a metastatic pulmonary LCNEC during intraoperative pathological evaluation.

## Case presentation

A 69-year-old woman with a 20-pack-year smoking history presented with progressive anorexia and significant weight loss over several weeks. Her referring physician observed mild response latency during the initial evaluation and ordered a brain computed tomography (CT) scan in addition to chest and abdominal imaging.

**Figure 1:**
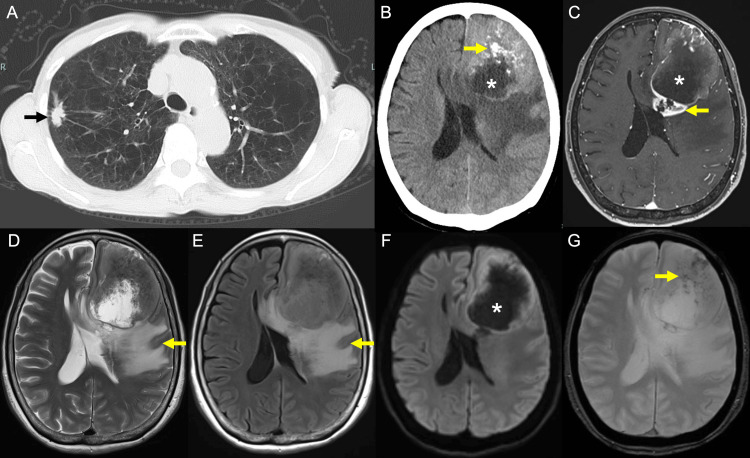
Preoperative CT and MR images (A) Chest CT demonstrates a solitary pulmonary nodule in the right upper lobe (arrow). (B) Non-contrast head CT shows a large, heterogeneous mass in the left frontal lobe measuring approximately 8 cm in diameter, with central coarse granular calcifications (arrow) and central hypodensity suggestive of necrosis (asterisk). (C) Brain MRI demonstrates a heterogeneously enhancing mass with peripheral ring enhancement (arrow) and a non-enhancing necrotic core (asterisk) on post-contrast T1-weighted imaging. (D, E) T2-weighted and fluid-attenuated inversion recovery (FLAIR) sequences demonstrate extensive perilesional hyperintensity, consistent with vasogenic edema (arrows in D and E). (F) Diffusion-weighted imaging (DWI) shows low signal intensity in the necrotic core (asterisk) without restricted diffusion at the enhancing rim. (G) T2*-weighted gradient-echo (GRE) sequences reveal intralesional signal voids suggestive of calcification and/or hemorrhage (arrow).

Chest CT revealed a solitary pulmonary nodule in the right upper lobe (Figure 1A). Laboratory testing showed a markedly elevated progastrin-releasing peptide (proGRP) level (>1500 pg/mL), suggesting a neuroendocrine neoplasm. A non-contrast head CT revealed a large, heterogeneous mass in the left frontal lobe, measuring approximately 8 cm in diameter, with central coarse granular calcifications and central hypodensity suggestive of necrosis (Figure 1B). The lesion exerted mass effect, compressing the anterior horn of the left lateral ventricle and causing a moderate midline shift.

Neurological examination revealed mild aphasia and subtle right-sided hemiparesis. No seizures or visual disturbances were observed. MRI of the brain demonstrated a heterogeneously enhancing mass with peripheral ring enhancement and a non-enhancing necrotic core on post-contrast T1-weighted images (Figure 1C). The medial margin was relatively smooth and adjacent to the lateral ventricle, whereas the lateral and posterior borders were irregular, raising concerns about infiltrative growth. T2-weighted and FLAIR sequences demonstrated extensive perilesional hyperintensity, consistent with vasogenic edema (Figures 1D, 1E). DWI revealed low signal intensity in the necrotic core without restricted diffusion at the enhancing rim (Figure 1F). T2*-weighted GRE sequences showed intralesional signal voids, suggestive of calcification and/or hemorrhage (Figure 1G).

Based on imaging and laboratory findings, the working diagnosis was lung cancer, most likely small-cell lung carcinoma (SCLC), with synchronous brain metastasis. High-grade gliomas, such as glioblastomas, remained a significant differential consideration owing to the lesion’s location and the presence of calcification, a characteristic feature of gliomas.

**Figure 2:**
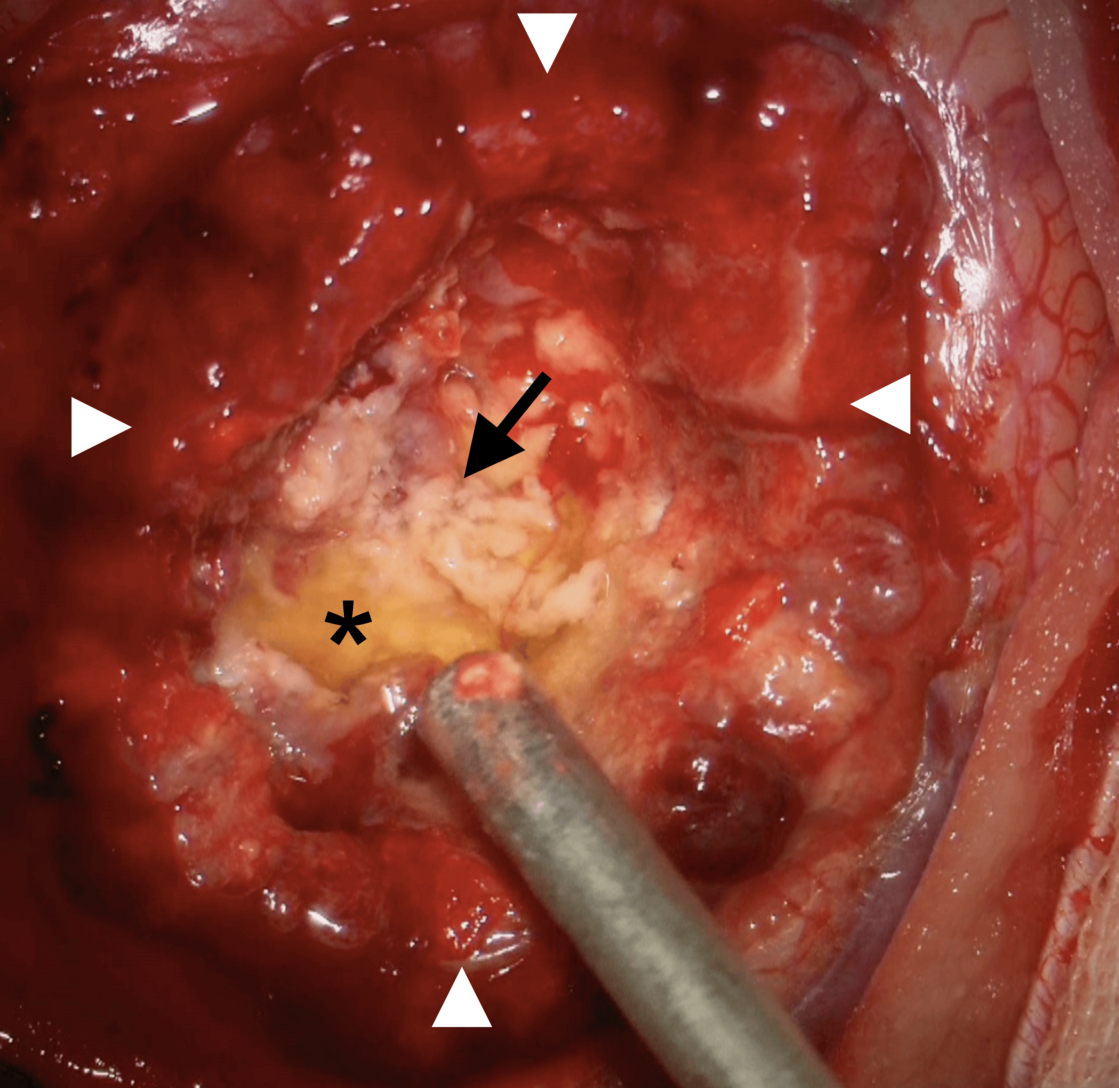
Intraoperative microscopic view Intraoperative microscopic view after partial removal, showing a moderately vascular area (white arrowheads), regions of necrosis (arrow), and fluid collection (asterisk).

The patient underwent a left frontal craniotomy and microsurgical resection. Intraoperatively, the tumor was located immediately beneath the dura, appeared partially calcified, was moderately vascular, and contained areas of necrosis and fluid (Figure 2). Considering the intraoperative frozen section diagnosis of metastatic carcinoma rather than glioma, the resection was limited to the gadolinium-enhancing lesion, leaving the T2/FLAIR hyperintense region unresected.

**Figure 3:**
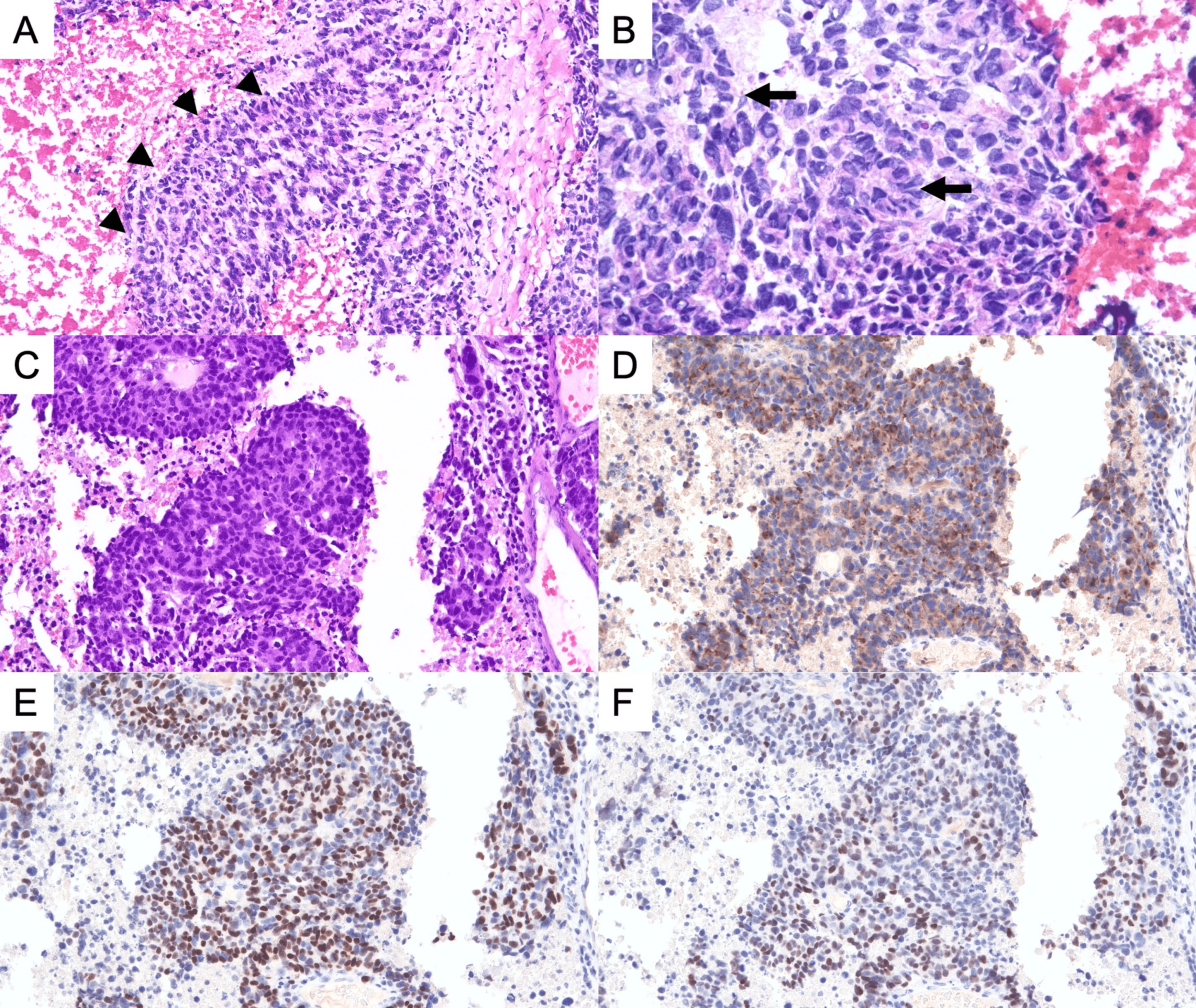
Pathology (A, B) H&E shows large atypical epithelioid cells proliferate with nuclear palisading (arrowheads in A) and rosette-like structure (arrows in B) in the intraoperative frozen section. (C) In the permanent section, these pathognomonic features are confirmed. (D–F) An immunohistochemical study shows positive staining for chromogranin A (D), INSM1 (insulinoma-associated protein 1) (E), and TTF-1 (thyroid transcription factor-1) (F). Images were taken at 200× (A, C–F) and 400× (B). H&E: hematoxylin and eosin

Intraoperative frozen sections revealed tumor tissue with prominent necrosis and proliferating atypical large cells exhibiting a cohesive, epithelial-like architecture. Palisading patterns, rosette-like structures, and nuclear palisading were observed in non-necrotic areas (Figures 3A, 3B). Based on these findings, pulmonary LCNEC was considered the primary diagnostic possibility.

The final histopathological examination revealed proliferation of atypical large epithelial cells with extensive necrosis. Nuclear palisading and rosette formation were observed (Figure 3C). Although the cells exhibited a high nuclear-to-cytoplasmic (N/C) ratio and inconspicuous nucleoli, the cytoplasm was more prominent than typically seen in small-cell carcinoma, and the nuclei lacked the characteristic finely granular ("salt-and-pepper") chromatin pattern. Immunohistochemical staining was positive for chromogranin A (Figure 3D), INSM1 (insulinoma-associated protein 1) (Figure 3E), and TTF-1 (thyroid transcription factor-1) (Figure 3F). Overall, the histological features were consistent with those of LCNEC and interpreted as metastasis from a primary pulmonary tumor.

The postoperative course was uneventful. The patient’s aphasia and right hemiparesis improved following surgery. She subsequently underwent adjuvant radiotherapy, and systemic chemotherapy was planned under the care of the pulmonary department.

## Discussion

LCNEC is a rare, high-grade pulmonary malignancy that exhibits overlapping features of both small-cell lung carcinoma and non-small cell lung carcinoma [1]. Brain metastases are often the initial manifestation, particularly in asymptomatic patients. In a multicenter study, 40.3% of patients with LCNEC presented with synchronous brain metastases at the time of initial diagnosis [2]. Similarly, a population-based analysis reported that 23% of patients with stage IV LCNEC had brain metastases at presentation [3].

In the present case, the imaging findings posed a diagnostic dilemma. Coarse granular calcification of the lesion and its frontal location suggested oligodendroglioma, whereas the ring enhancement pattern, central necrosis, and relatively well-circumscribed margins were consistent with metastatic disease. The key clinical clues favoring metastatic pulmonary neuroendocrine carcinoma were the solitary pulmonary nodule and markedly elevated serum proGRP level; however, these findings did not completely exclude glioma because the frontal location and coarse calcification remained compatible with oligodendroglioma.

CBMs have traditionally been considered rare; however, recent CT-based studies suggest that they may be more common than previously recognized. Prior reports have described calcified metastases from various primary tumors, including lung cancer and neuroendocrine neoplasms, highlighting that calcification does not exclude metastatic disease. The present case supports these observations and further illustrates that LCNEC may present with prominent calcification, mimicking primary brain tumors. The incidence of CBMs in oncologic populations has been reported between 7.9% and 9.5% at initial presentation [4,5]. These lesions are most frequently associated with primary lung and breast adenocarcinomas. Calcification is also a well-documented radiographic feature of certain glioma subtypes. In particular, oligodendrogliomas (IDH-mutant, 1p/19q-codeleted) exhibit calcification in up to 36.7% of cases, as demonstrated in a retrospective series of 305 patients [6].

Histopathological assessment, in combination with imaging studies, plays an important role in establishing the diagnosis. Frozen sections revealing rosette-like structures and peripheral palisading are more suggestive of a neuroendocrine origin than a glial origin [7]. Although SCLC was an important differential diagnosis, calcification itself is not specific for LCNEC and has been reported in brain metastases from various primary tumors, including SCLC. In the present case, calcification mainly contributed to the radiological diagnostic dilemma by mimicking a calcified glioma rather than distinguishing LCNEC from SCLC. The diagnosis of LCNEC was favored based on the tumor morphology, including large atypical epithelial cells with relatively abundant cytoplasm, rosette-like structures, and nuclear palisading, together with positive immunohistochemical staining for neuroendocrine and pulmonary markers [4,7].

In this case, intraoperative histopathological assessment was instrumental in guiding the surgical strategy. In current neurosurgical practice, maximal safe resection is a key principle in glioma management [8-10]. However, this concept differs from the surgical strategy for brain metastases. For metastatic brain tumors, resection is generally limited to contrast-enhancing lesions, as non-enhancing FLAIR abnormalities usually represent vasogenic edema rather than tumor infiltration. Therefore, excessive resection beyond the enhancing margin is not recommended [11,12]. Based on the intraoperative frozen section diagnosis, resection in the present case was limited to the contrast-enhancing lesion, avoiding the FLAIR hyperintense peritumoral region.

This case report highlights several important points. First, it reinforces existing evidence that calcification does not exclude metastatic disease, even in solitary brain lesions. Second, it demonstrates that LCNEC can present with radiological features that closely mimic those of glioma, leading to diagnostic uncertainty. Third, it emphasizes the critical role of intraoperative pathological evaluation in guiding surgical decision-making, particularly in distinguishing between primary brain tumors and metastases. While this approach is well recognized, this case provides a practical example of its direct impact on surgical strategy.

To the best of our knowledge, this is one of the few reported cases of solitary CBM from LCNEC diagnosed intraoperatively. Early integration of radiological, pathological, and surgical data is essential for accurate diagnosis and optimal patient management.

## Conclusions

This case illustrates an unusual presentation of pulmonary LCNEC as a solitary, calcified frontal lobe metastasis. Its imaging features overlapped with those of primary brain tumors, particularly calcified gliomas, creating a diagnostic challenge. Calcification should not exclude metastatic disease from the differential diagnosis, even in patients without known malignancy or respiratory symptoms. Intraoperative histopathological evaluation may help clarify the diagnosis when imaging findings are inconclusive. As this report describes a single case, its primary contribution is educational and diagnostic rather than the establishment of broader clinical recommendations.
